# Age and Sport Intensity-Dependent Changes in Cytokines and Telomere Length in Elite Athletes

**DOI:** 10.3390/antiox10071035

**Published:** 2021-06-28

**Authors:** Maha Sellami, Shamma Al-muraikhy, Hend Al-Jaber, Hadaia Al-Amri, Layla Al-Mansoori, Nayef A. Mazloum, Francesco Donati, Francesco Botre, Mohamed A. Elrayess

**Affiliations:** 1 Department of Physical Education (PE), College of Education, Qatar University, Doha P.O. Box 2713, Qatar; msellami@qu.edu.qa; 2 Biomedical Research Center, Qatar University, Doha P.O. Box 2713, Qatar; salmuraikhy@qu.edu.qa (S.A.-m.); haljaber@qu.edu.qa (H.A.-J.); almansouri@qu.edu.qa (L.A.-M.); 3 Department of Sport Science Program (SSP), College of Arts and Science, Qatar University, Doha P.O. Box 2713, Qatar; ha1402415@student.qu.edu.qa; 4 Department of Microbiology and Immunology, Weill Cornell Medicine-Qatar, Doha P.O. Box 24811, Qatar; nam2016@qatar-med.cornell.edu; 5 Laboratorio Antidoping, Federazione Medico Sportiva Italiana, Viale Tiziano 70, 00196 Rome, Italy; donati.francesco@gmail.com (F.D.); botref@gmail.com (F.B.)

**Keywords:** aging, telomere length, elite athletes, anti-inflammatory cytokines, pro-inflammatory cytokines, sport intensity

## Abstract

Exercise-associated immune response plays a crucial role in the aging process. The aim of this study is to investigate the effect of sport intensity on cytokine levels, oxidative stress markers and telomere length in aging elite athletes. In this study, 80 blood samples from consenting elite athletes were collected for anti-doping analysis at an anti-doping laboratory in Italy (FMSI). Participants were divided into three groups according to their sport intensity: low-intensity skills and power sports (LI, *n* = 18); moderate-intensity mixed soccer players (MI, *n* = 31); and high-intensity endurance sports (HI, *n* = 31). Participants were also divided into two age groups: less than 25 (*n* = 45) and above 25 years old (*n* = 35). Serum levels of 10 pro and anti-inflammatory cytokines and two antioxidant enzymes were compared in age and sport intensity groups and telomere lengths were measured in their respective blood samples. Tumor necrosis factor-alpha (TNF-α) was the only cytokine showing significantly higher concentration in older athletes, regardless of sport intensity. Interleukin (IL)-10 increased significantly in HI regardless of age group, whereas IL-6 concentration was higher in the older HI athletes. IL-8 showed a significant interaction with sport intensity in different age groups. Overall, significant positive correlations among levels of IL-6, IL-10, IL-8 and TNF-α were identified. The antioxidant catalase activity was positively correlated with levels of TNF-α. Telomere length increased significantly with sport intensity, especially in the younger group. HI had longer telomeres and higher levels of pro- and anti-inflammatory cytokines, suggesting less aging in HI compared to low and moderate counterparts in association with heightened immune response. Investigation of the functional significance of these associations on the health and performance of elite athletes is warranted.

## 1. Introduction

The past decade has witnessed an increased interest in the aging of the immune system. With aging, immune cells decrease in number and undergo impaired functioning, which reduces their efficiency and leads to a chronic inflammatory state [1]. The health benefits of physical activity on biological systems are well established. However, the effect of physical activity on the aging of the immune system and the underlying regulators are not well characterized. Improving immunological factors through exercise might exert an anti-immunosenescence effect [2,3]. 

Advanced age is accompanied by a chronic low-grade systemic inflammatory condition called “inflamm-aging” [4]. It is characterized by the increased production of proinflammatory cytokines such as interleukin (IL)-1, IL-6, “tumor necrosis factor alpha” (TNF-α) as well as by the production of C-reactive protein (CRP) [4]. The level of this proinflammatory state is associated with a poorer prognosis in elderly patients with increased risk of morbidity and mortality, sarcopenia and frailty [5]. Despite its importance, the precise cellular and molecular mechanisms leading to these alterations are not elucidated, but several hypotheses have been highlighted, including oxidative stress, persistent DNA damage, the aging of stem cells and the inhibition of autophagy through the activation of the inflammasome [6]. Conversely, older patients with high levels of anti-inflammatory cytokines seem to have a healthy aging status compared to others. Thus, a high level of IL-10 is associated with a lower risk of cardiovascular mortality [7] and is found more frequently among older people [8].

Regular practice of physical activity can induce long-term adaptations in the metabolic and cardiovascular system. These include decreased reactive oxygen species (ROS) and pro-inflammatory cytokines with a concomitant rise in antioxidants and anti-inflammatory factors [9]. The analysis of the functional interrelationships between physical exercise and the immune system is extremely complex due to the multiplicity of underlying mediators (type of exercise, frequency, and intensity) and the individual variability of the immune response to exercise-induced stress [10]. Stewart et al. [11] found that a 12-week aerobic exercise training did not alter plasma IL-6 and IL-1β when comparing young groups of 18–35-year-olds to older ones, suggesting that short-term training (months) may not be sufficient to induce changes in proinflammatory cytokines. Additionally, Barry et al. [12] demonstrated that short-term (2 weeks) high-intensity interval training and moderate-intensity training did not change circulating levels of IL-10, IL-6, or TNF-α in obese individuals. Conversely, findings from our previous study [13] suggested that cytokine levels vary significantly among athletes who belong to different sport disciplines.

Previous studies have shown that the age-dependent pro-inflammatory or oxidative cellular environment could promote telomere attrition, cellular senescence and disease [14]. However, a healthy diet and exercise training were shown to reduce the rate of telomere shortening during the aging process [15,16]. Studies have also shown that regular engagement in exercise training is associated with longer telomeres and may attenuate telomere attrition [17]. The majority of studies investigating the training effect on telomere length (TL) were based on questionnaire assessment of physical activity [18]. However, the optimal exercise recommendations for TL maintenance remain elusive. Accordingly, it would be of interest to study the TL in different sport intensities in relation to circulating inflammatory cytokine levels and the role of the antioxidants to understand the interplay between sport intensity, immune response and aging.

## 2. Materials and Methods

### 2.1. Cohort

Elite athletes in this study are defined as athletes who participated in national or international sport events and had their samples tested for doping abuse by anti-doping laboratories. Blood samples from 80 consenting elite athletes (14 females and 66 males, 26.4 ± 7.6 years old) were previously collected by a doping officer in serum separator tubes for human growth hormone at an anti-doping laboratory. Once collected, samples were delivered to the anti-doping labs within 36 h under cooling conditions. Once received, samples were immediately centrifuged to separate the serum and then stored at −20 °C until analysis. Inclusion criteria included athletes (males and females) whose samples tested negative for growth hormone at an anti-doping laboratory in Italy (FMSI). Exclusion criteria included athletes whose samples tested positive for any doping abuse. Due to the strict anonymization process undertaken by the anti-doping laboratories and as per the study’s ethics, only information related to the type of sport and athlete’s gender and age was available to researchers. Twenty athletes were tested “in competition” and 60 “out of competition”. Samples (fresh aliquots) were transported on dry ice to Qatar University and stored at −80 °C degrees until further analysis. Following published criteria [19,20], participants were divided into three intensity groups based on maximum oxygen uptake (VO2max) of their respective sports. Accordingly, participants were divided into low-intensity skills and power sports that require lower VO2max (LI, *n* = 18, age: 25 ± 8.3 years old), moderate-intensity soccer that requires moderate VO2max (MI, *n* = 31, age: 28.0 ± 7.4 years old), and high-intensity endurance sports that require high VO2max (HI, *n* = 31, age: 26.4 + 8.5 years old, including 12 athletes, 11 cyclists and 8 triathletes) (Table 1). According to age, participants were divided into two groups: less than 25 years old and above 25 years old. This study is performed in line with the World Medical Association Declaration of Helsinki—Ethical Principles for Medical Research Involving Human Subjects. All protocols were approved by the Institutional Research Board of Qatar University (QU-IRB 1277-E/20).

### 2.2. Cytokine Profiling

The Human Corplex Cytokine Panel 1 10-Plex Array was used to simultaneously profile 10 cytokines including IL-12p70, IL-1β, IL-4, IL-5, IFNγ, IL-6, IL-8, IL-22, TNF-α, IL-10 using Quaterix (Human 10-Plex 116-7BF-1-AB) in the sera of 80 samples described in Table 1, according to manufacturer’s instructions (Quanterix Inc, Billerica MA, USA). Briefly, samples were diluted 1:4 in sample diluent. Diluted samples and calibrators were added to 96-well microplates pre-spotted with protein-specific antibodies and incubated for 2 h at room temperature. Biotinylated antibody reagent was added and incubated for 30 min at room temperature, followed by addition of Streptavidin–Horseradish Peroxidase (HRP) Reagent A and incubation for 30 min at room temperature. After washing, SuperSignal^®^ Substrate was added and the luminescence was read using SP-X Imaging System (Quanterix, USA). Separate standard curves were used to validate the assay for the detection and quantification of cytokines according to manufacturer’s instructions. Sensitivity and selectivity can be found at manufacturer’s website (https://www.thermofisher.com/document-connect/document-connect.html?url=https://assets.thermofisher.com/TFS-Assets/LSG/manuals/MAN0017197_HumanCytokineMagnetic10PlexPanel_TDS.pdf, accessed on 25 June 2021).

### 2.3. Measurement of Antioxidant Enzymatic Activities

Activities of superoxide dismutase and catalase were determined in the sera of 80 samples described in Table 1 using colorimetric activity assays (EIACATC and EIASODC, respectively), according to manufacturer’s instructions (ThermoFisher Scientific, Fredrick, MD, USA). Briefly, samples were diluted 1:5 in assay buffer. For measurement of catalase activities, diluted samples and catalase standards were added to hydrogen peroxide reagent in 96-well plates and incubated for 30 min, followed by addition of substrate and HRP solution and incubation for 15 min at room temperature. Absorbance was then read at 560 nm using Cell Imaging Multi-Mode Reader (BioTek, Agilent, Winooski, VT, USA). For measurement of superoxide dismutase activity, diluted samples and SOD standards were added to the substrate in 96-well plates. Chromogenic detection reagent (Xanthine Oxidase) was then added and incubated for 20 min at room temperature, followed by measuring absorbance at 450 nm by Cell Imaging Multi-Mode Reader (Agilent).

### 2.4. Measurement of Telomere Length

PureLink^®^ Genomic DNA Kits (Invitrogen, Life Technologies, Carlsbad, CA, USA) were used for isolation of genomic DNA from the clotted blood at the bottom of the serum tubes from 80 samples described in Table 1, according to manufacturer’s instructions. The concentration and the quality of DNA were measured using the Nanodrop. Absolute Human Telomere Length Quantification qPCR Assay Kit (ScienCell, Carlsbad, CA, USA) was used to measure the average telomere length in extracted DNA samples according to manufacturer’s instructions. The kit includes a telomere primer set that amplifies telomere sequences, a single copy reference region for data normalization and a reference genomic DNA sample with known telomere length as a reference for calculating the telomere length of target samples. Briefly, two qPCR reactions were prepared for each genomic DNA sample: one with telomere (TL) and one with single copy reference (SCR) primer stock solutions. qPCR reactions were prepared by adding genomic DNA template (5 ng/µL) to primer stock solution (TL or SCR) and GoldNStart TaqGreen qPCR master mix. qPCR was run using an initial denaturation of 95 °C for 10 min, followed by 32 cycles of denaturation at 95 °C for 20 s, annealing at 52 °C for 20 s and extension at 72 °C for 45 s using StepOne™ Real-Time PCR System (ThermoFisher). For quantification of TL, ∆Cq (TL) was quantified by assessing the cycle number difference of TL between two genomic DNA samples (sample of interest and the reference genomic DNA sample with known telomere length). For SCR, ∆Cq (SCR) was assessed by quantifying the cycle number difference of SCR between two genomic DNA samples (sample of interest and the reference genomic DNA sample with known telomere length). ∆∆Cq was calculated as ∆Cq (TL)−∆Cq (SCR). Fold change was assessed as 2-∆∆Cq.

### 2.5. Statistical Analysis

Comparisons were performed using *t*-test, Wilcoxon–Mann–Whitney, 1-way ANOVA, or linear models as appropriate using IBM SPSS statistics 21. Linear regression models were used when analyzing differences in cytokine levels among different age and intensity groups by considering gender and in/out of competition as potential confounders. Data were presented as mean ± standard deviation (SD) for parametric data and median (interquartile range, IQR) for non-parametric data, verified by skewness and kurtosis test. Based on our previous findings [13] that showed an average effect size of 36% of endurance on cytokine levels with 40% variation, power calculations indicated that the current sample size (*n* = 80) had 95% power to detect a minimal difference of 36% in cytokine levels with 40% deviation from mean value (σ) at a level of α = 0.01.

## 3. Results

**Figure 1:**
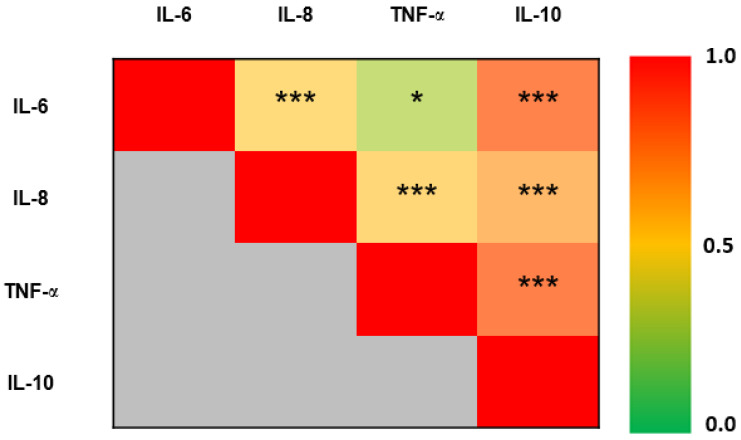
Heatmap representing significant positive correlations among cytokines measured in the whole cohort (*n* = 80). Correlation coefficient range (0–1) is color-coded (green to red). Correlations were made using Spearman’s correlation analysis (* *p* ≤ 0.05, *** *p* ≤ 0.001).

Out of the 10 measured cytokines, only results from four cytokines are reported in 80 elite athletes, namely, IL-6, IL-8, TNF-α and IL-10. The remaining cytokines were either below the detection limit of the kit (IL-12p70, IL-5, IFNγ) or did not show significant differences by age or intensity groups (IL-1β, IL-4, IL-22). Table 2 summarizes the results comparing levels of detected cytokines by age and intensity groups. There was a significant interaction between age and intensity with regard to IL-6 levels. Significant differences were observed by intensity between the two age groups, as IL-6 levels were higher in moderate intensity in athletes under 25 years old compared to lower intensity counterparts, whereas levels were not different between low- and moderate-intensity groups in athletes above 25 years old. A similar interaction between age and intensity groups was observed with regard to IL-8, as levels decreased with high intensity in athletes under 25 years old, whereas levels increased in high intensity in those above 25 years. TNF-α levels varied significantly with age within the intensity groups, as they decreased with increased intensity in athletes under 25 years old and they decreased with intensity in the above 25 years old counterparts. In total, TNF-α levels were higher in athletes above 25 years old than their younger counterparts. Similarly, IL-10 also showed significant differences within intensity groups by age, as levels did not change in athletes under 25 years old, whereas levels were reduced in moderate athletes above 25 years old. Finally, telomere lengths also varied significantly by intensity in different age groups, as they were higher in moderate and high intensities in athletes under 25 years old, whereas they were higher only in high-intensity groups in those above 25 years old. There were significant positive correlations among levels of IL-6, IL-10, IL-8 and TNF-α (Figure 1).

**Figure 2:**
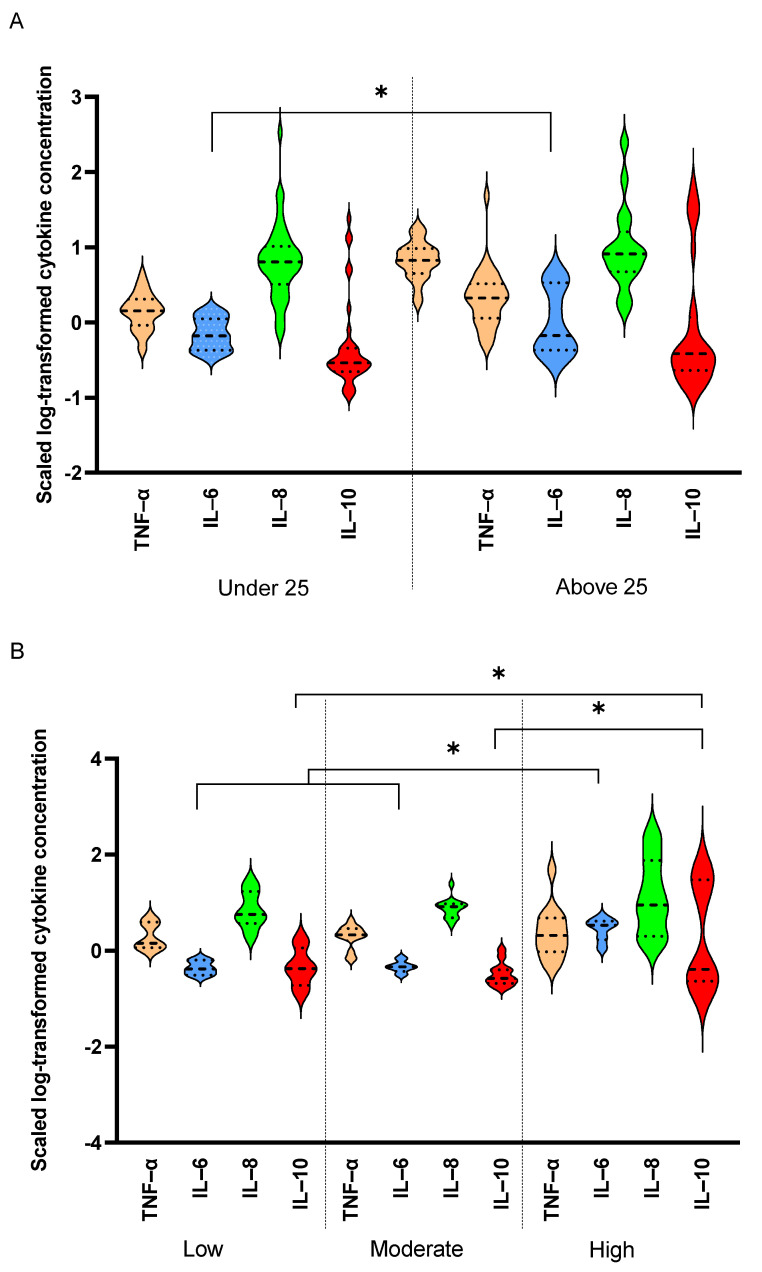
Comparing levels of serum cytokines between age and sport intensity groups. IL-8 and IL-10 were compared between younger (less than 25 years old, *n* = 45) and older (more than 25 years old, *n* = 35) elite athletes (**A**) in different sport intensities (low, *n* = 18, moderate, *n* = 31 and high, *n* = 31) (**B**). ANOVA was used to compare cytokine levels in different age and sport intensity groups. Data are presented as median and IQR. p* <0.05.

IL-6 and IL-10 were elevated in higher intensity sports in an age-dependent manner: When comparing levels of detected cytokines among different sport intensities, there were significant elevations in IL-6 (*p* = 0.02) and IL-10 (*p* = 0.03) with increased sport intensity (Figure 2B). No significant differences were detected in the levels of TNF-α and IL-8 with increased sport intensity, although a non-significant trend was seen in IL-8 levels (*p* = 0.1).

**Figure 3:**
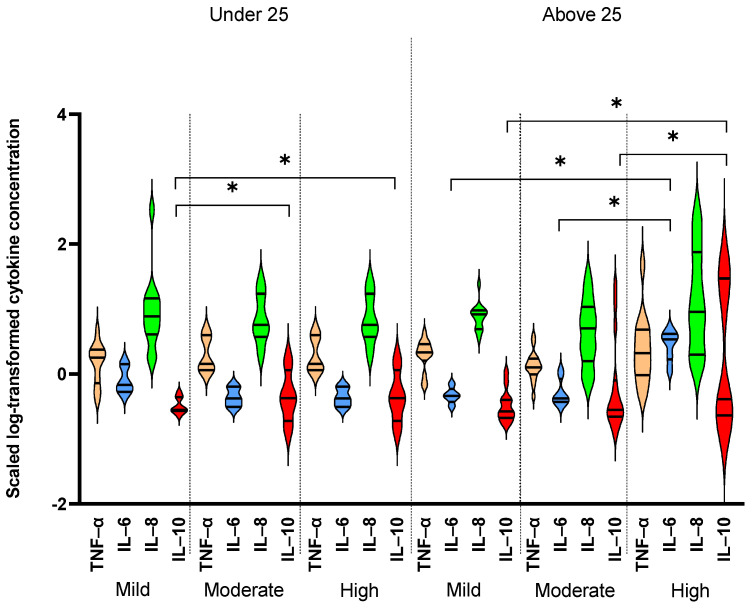
Comparing levels of serum cytokines between age and sport intensity groups. TNF-α, IL-6, IL-8 and IL-10 were compared between younger (less than 25 years old, *n* = 45) and older (more than 25 years old, *n* = 35) elite athletes in different sport intensities (low, *n* = 18, moderate, *n* = 31 and high, *n* = 31). Linear regression model was used to compare cytokine levels in different age and sport intensity groups taking gender and in/out of competition status as potential confounders. Data are presented as median and IQR. * *p* < 0.05.

There was a significant difference in the levels of IL-6 and IL-10 among different sport intensities between the two age groups (Table 2, Figure 3). In this regard, IL-10 showed a significant elevation in younger athletes who belong to moderate- and high-intensity sports compared to low-intensity counterparts, whereas it only exhibited a significant increase in older athletes who belong to high-intensity sports compared to low/moderate counterparts (Figure 3). On the other hand, IL-6 showed a significant increase in the high-intensity sport group compared to low/moderate counterparts only in the older group (Figure 3).

**Figure 4:**
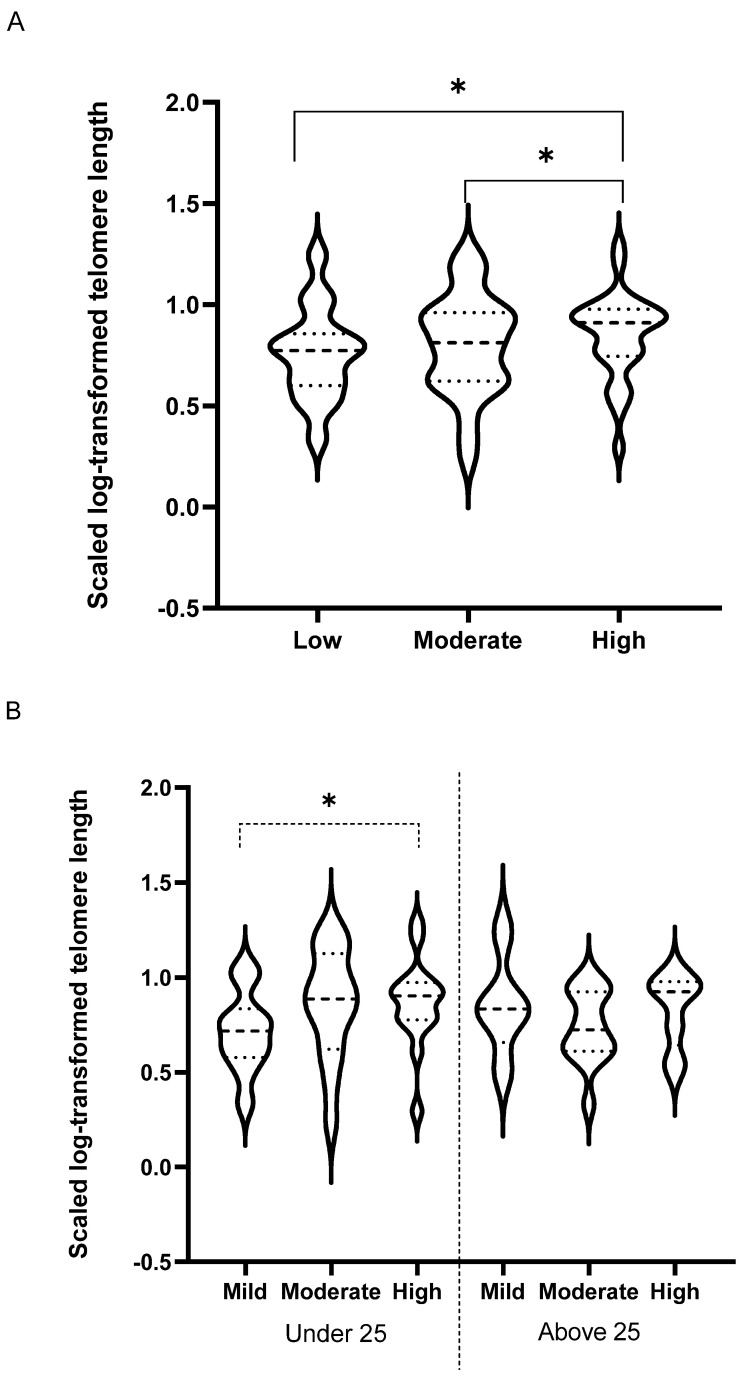
Comparing telomere length between age and sport intensity groups. Telomere lengths were compared between younger (less than 25 years old, *n* = 45) and older (more than 25 years old, *n* = 35) elite athletes (**A**) in different sport intensities (low, *n* = 18, moderate, *n* = 31 and high, *n* = 31) (**B**). Linear regression model was used to compare cytokine levels in different age and sport intensity groups taking gender and in/out of competition status as potential confounders. Data are presented as median and IQR. * *p* < 0.05.

High-intensity sports were associated with longer telomere length (TL): When comparing telomere length in elite athletes who belong to different age and intensity groups, the results indicated no significant difference in TL between the two age groups (Table 2). However, the results revealed a significant increase in TL in athletes who belong to high-intensity sports compared to low and moderate sport intensity groups (*p* = 0.03) (Figure 4A). This difference was significant in younger (*p* = 0.03) but not older elite athletes, although a similar trend was detected (*p* = 0.1) (Figure 4B).

**Figure 5:**
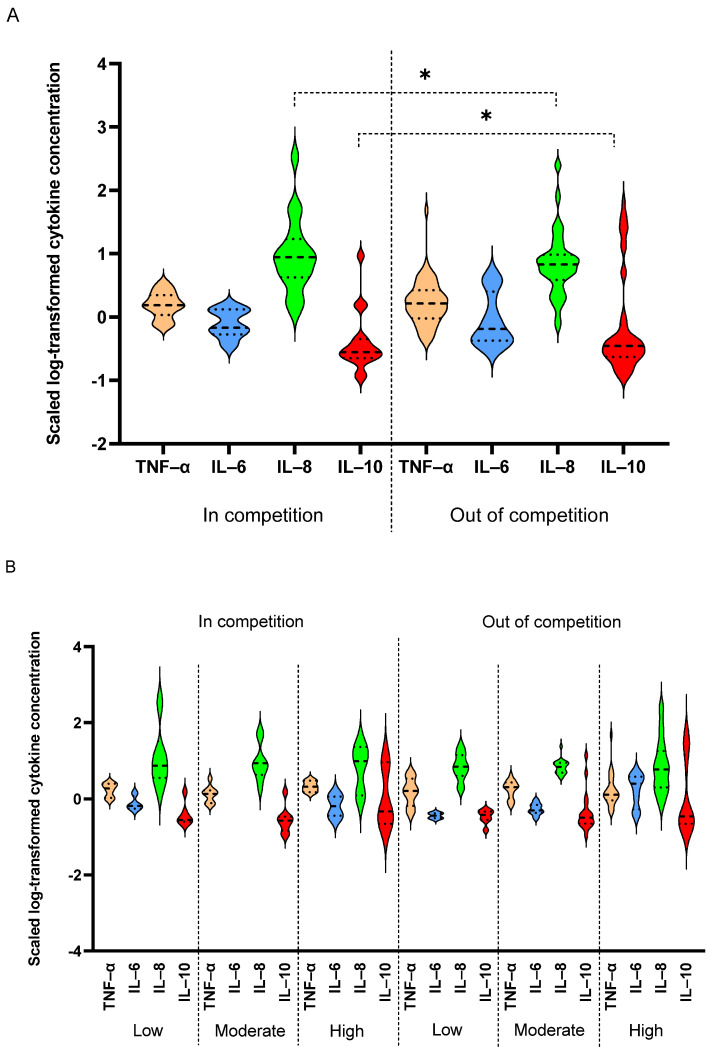
Comparing levels of cytokines in competing and out of competition athletes. Levels of TNF-α, IL-6, IL-8 and IL-10 cytokines were compared between in competition (*n* = 20) and out of competition (*n* = 60) elite athletes (**A**) in different sport intensities (low, *n* = 18, moderate, *n* = 31 and high, *n* = 31) (**B**). Linear regression model was used to compare cytokine levels in different age and sport intensity groups taking gender and in/out of competition status as potential confounders. Data are presented as median and IQR. * *p* < 0.05.

IL-6 and IL-10 were higher in competing athletes compared to out of competition counterparts: Since competition adds to athletes’ physiological and psychological stress, levels of cytokines were compared between competing (*n* = 20) and out of competition (*n* = 60) elite athletes. Results indicated higher levels of IL-6 and IL-10 in the competing athletes compared to the non-competing counterparts, regardless of age (*p* = 0.008 and *p* < 0.001, respectively) (Figure 5A). IL-8 was also higher in competing athletes compared to non-competing counterparts, but the difference in IL-8 levels did not reach statistical significance (*p* = 0.1). However, when considering sport intensity groups, there was a significant increase in IL-8 levels in competing athletes with increased sports intensity (*p* = 0.03), but not in non-competing athletes. A similar trend was also seen in TNF-α, IL-6 and IL-10, but did not reach statistical significance (Figure 5B).

Catalase activity was positively associated with levels of TNF-α: Levels of SOD and catalase did not differ between age or intensity groups (Table 2). However, there was a significant positive association between catalase activity and levels of TNF-α (R = 0.51, *p* = 0.003).

## 4. Discussion

Previous studies have reported that aging is associated with elevation in the pro-inflammatory environment, which promotes the shortening of telomeres, cellular senescence and health decline. The positive effect of exercise on modulating age-related inflammatory response is well established. However, studies investigating the effect of different levels of exercise intensity in athletes of different age groups on levels of cytokines and TL are not yet established. In this study, serum levels of various pro and anti-inflammatory cytokines and antioxidant enzymes were determined in elite athletes who belong to three different sport intensities and two age groups. Our emerging novel data show that HI sports were associated with longer telomeres and higher levels of pro- (IL-6) and anti-inflammatory (IL-10) cytokines, suggesting slower aging in HI sports accompanied with heightened inflammatory response.

Aging is associated with an elevation in blood inflammatory markers, including TNF-α [21] and IL-6 [22]. The aging-dependent increase in inflammatory response in older individuals could reflect health decline and disease progression. In our study, TNF-α was the only cytokine exhibiting a significant elevation in older elite athletes among all detected cytokines, regardless of intensity groups. Levels of TNF-α were also positively correlated with catalase activity, shown previously to be down-regulated by TNF-α [23]. Since the elite athletes participating in this study are relatively young compared to previous studies focusing on an aged population, our data may suggest that TNF-α could be used as an early biomarker of pro-inflammatory aging in elite athletes.

During a strenuous workout, numerous cytokines are released into circulation. Evidence from different cross-sectional studies found that levels of IL-6 can rise up to 100-fold and TNF-α by threefold over their baseline values [24]. Other studies have shown that exercise can increase IL-1β [25] and TNF-α [26], while others have found no changes in these cytokines [27]. Most of the previous findings explored the response of cytokines to long and continuous strenuous exercise such as a marathon, which makes extrapolation of the findings in low-intensity exercises difficult. In this study, the profiling of cytokines investigated elite athletes who belong to different age and sport intensity groups. Our data indicated that both IL-6 and IL-10 are elevated in HI sports in an age-dependent manner. Hence, levels of IL-6 and IL-10 were correlated with exercise intensity, suggesting that sports with more intense exercise can lead to increased production of these cytokines. The elevation in the anti-inflammatory cytokine IL-10 together with IL-6 could reflect a compensatory mechanism and adaptation to long-term training. Indeed, the concomitant increase in IL-6 and IL-10 in HI athletes seems to express a balance in pro- and anti-inflammatory processes as an adaptation for long-term exposure to stressful stimuli such as athletics and cycling sports. Westendorp [28] demonstrated that such a balance would be in favor of successful aging and longer life. Similarly, the correction between levels of TNF-α and IL-10 is important for maintaining immune homeostasis. The production of TNF-α contributes to overwhelming inflammatory response and tissue damage. However, the increase in TNF-alpha production is usually counterbalanced by simultaneous synthesis of an anti-inflammatory cytokine IL-10, which suppresses the production of many activating and regulatory mediators.

Interestingly, IL-10 was elevated in athletes who belong to HI sports in both age groups, and IL-6 was only elevated in older HI participants, but not in athletes below 25 years of age. The protective effect of IL-10 with HI sports seems to be independent of age, whereas the harmful effect of IL-6 is more manifested in older athletes. Our data also showed that IL-6 and IL-10 were higher in competing athletes compared to out of competition counterparts regardless of age. IL-8 showed higher levels with increased sport intensity only in competing athletes. Elevation in these cytokines during competition could reflect the effect of physiological and psychological stress due to increased intensity and frequency of exercise during competition and associated anxiety [29]. Furthermore, our data showed a clear difference in IL-6, but not other proinflammatory cytokines, among different intensity groups. This could reflect the sensitivity of IL-6 response to exercise, as reported by many studies [30,31,32,33]. IL-6 is an inflammation-responsive muscle-derived factor that signals to other organs during exercise. Previous studies have shown that subjects performing exercise in a controlled environment (exhaustive exercise and low in carbohydrates diet) exhibit elevated plasma levels of IL-6 released from their stressed muscles, especially in subjects with lower muscle glycogen stores before exercise [24]. Such results may explain the source of the higher IL-6 in the HI group that is essentially composed of athletes who belong to athletics and cycling sports. An interesting study of Bourne and Rapoport [34] showed that marathon athletes can “hit the wall” by reducing glycogen stores below their optimal levels. Therefore, as glycogen continues to decline during intense long exercises, IL-6 continues to rise [35]. Other findings suggest that exposure to higher proinflammatory IL-6 can trigger an increase in glycogen stores and a change in insulin sensitivity [36]. This may in part explain the physiological adaptation of marathon runners and elite cyclists, as they need to have adequate glycogen stores before competitions.

On other hand, anti-inflammatory cytokines, such as IL-10, appear later in exercise or during recovery as a compensatory mechanism to counteract the rising pro-inflammatory cytokines during a workout [37]. Among anti-inflammatory cytokines, IL-10 is arguably the most potent immunosuppressive cytokine. It can block immune responses at different levels; therefore, it is likely to play an important role in the control of immune and inflammatory reactions during exercise. In fact, IL-10 showed highly significant correlations with levels of IL-6, IL-8 and TNF-α, which may explain the compensatory mechanism in the HI sport group. Indeed, it was well demonstrated that IL-10 can block the release of IL-1α, IL-1β, TNF-α and IL-8 [38]. Pro- or anti-inflammatory cytokines have a role in all phases of inflammation. It is the balance between pro and anti-inflammatory cytokines that will locally manage the intensity and duration of the inflammatory reaction induced by physical activity [39]. Previous studies have demonstrated that HI exercise training/increased intensity is prone to a higher risk of injury [40]. Our findings, however, suggest a new theory of a balanced inflammatory status that argues against the negative impact of stressful workouts. Further research is required to explore whether such a balanced inflammatory status could be supported and to test the two emergent biomarkers (IL-6 and IL-10) as indicators for successful training-associated reduced aging.

Telomeres are regions of repetitive nucleotide sequences at the ends of chromosomes, which act as a protective layer to prevent the loss of genetic information through degradation. However, with the natural aging process, the DNA length inevitably decreases with each cell division until it reaches a critical length. The decrease in TL is considered a strong cellular indicator of biological aging and potentially health decline. Different modes of acute exercise might be able to affect TL and increase the health status and lifespan of a person [41,42]. Previous studies have tested the effect of 6 months of aerobic endurance training, high-intensity training, and resistance training in 124 healthy, but inactive, individuals. The results from these studies indicated that endurance, but not resistance, training acutely increased telomerase activity and TL [43]. Other studies reported a similar response to acute exercise, proposing an adaptive mechanism that might contribute to TL maintenance [42]. Two large datasets from the US National Health and Nutrition Examination Survey have shown that longer telomeres are associated with increased survival, particularly among active men [44,45]. A cross-sectional study of 7813 women aged 43–70 years from the Nurses’ Health Study has shown that even moderate amounts of activity may be associated with longer telomeres [46]. Studies have also pointed to sex differences in anti-aging response to short- and long-term high-intensity interval exercise related to telomerase activity and total oxidative stress status [47], and suggested that decreasing oxidative stress and inflammation could protect people from telomere attrition [48]. Recent evidence has suggested that young elite athletes have longer telomeres than their inactive peers [43]. A meta-analysis comparing the effect of high levels of physical activity in elite athletes compared to a sedentary lifestyle on telomere length concluded that high-level chronic physical training may provide protective effects on TL [49]. Among endurance athletes, a strong correlation between maximum oxygen uptake and the ratio of telomeres/single copy genes was identified [50]. Furthermore, a recent study has suggested that the longer leukocyte TL of master athletes may reflect a better ability to face COVID-19 successfully than their frail sedentary age-matched peers [51]. Longer telomeres of master athletes compared to age-matched non-athletes may be the result of lower chronic inflammation and oxidative stress and elevated telomerase activity [52]. In contrast, other reports proposed that exposure to acute endurance exercise might be threatening to an individual’s health as it leads to accelerated oxygen radical generation and oxidative stress [53,54]. Acute exposure to long distance running was also found to decrease TL due to oxidative DNA damage. Reports have shown that although antioxidant enzyme activities were increased following long-term exercise training, the lengths of telomeres in leukocytes were not influenced by both mid-intensity and high-intensity exercise stress [55]. Therefore, evidence of the effect of exercise on TL remains controversial and warrants further investigation. Our study, therefore, aimed to clarify the effect of the chronic effect of different sport intensities in elite athletes on TL and potentially cellular aging. Our novel data indicated a significant increase in TL in athletes who belong to HI sports. The effect was more pronounced in younger, but not older, elite athletes, although a similar trend was detected in older athletes. Hence, our data confirm previous reports of the positive effect of exercise on TL and reduced aging. The effect was more evident in younger athletes, perhaps as the effect of IL-6 elevation with increased sport intensity is less pronounced compared to older athletes. IL-6 is known to be one of the main signaling pathways involved in chronic morbidity and aging [30]. Therefore, our data are the first to confirm the maintenance of telomere length in highly trained athletes who belong to high-intensity sports.

This study presents some limitations that should be acknowledged. One of the main limitations of this study is the relatively small size of the studied groups. Additionally, variation in sample collection, transportation and storage could have affected the results. Furthermore, the recruitment timing (in or out of competition) has introduced another factor of stress-related alterations in cytokine levels, potentially confounding our data, although we have taken it into account during analysis. Although various studies have investigated the effect of acute exercise on cytokine levels [30,56,57,58], the use of samples from competing athletes under acute exercise could have impaired the interpretation of our results. However, by comparing changes in cytokines between athletes in and out of competition, the emerging data have provided crucial information about differences in cytokine levels between acute and chronic exercise periods. Furthermore, measuring antioxidant enzyme activities in the whole blood instead of athletes’ sera would have provided more relevant information; however, as samples were collected in serum separator tubes as per doping analysis protocols, it was not possible to perform these tests in whole blood samples. Finally, the limited available information of athletes’ anthropometric, physiological and nutritional data during sampling as well as the resting time since their last exercise has hindered attempts to consider other important potential confounders such as body mass index, VO2max and diet in our analysis. The missing information could have significantly influenced the inflammatory state of the athletes and the true correlation with anaerobic or aerobic exercise. Despite these limitations, our novel data revealed significant and pronounced alterations in cytokines and TL in the studied groups, although the replication of data in other cohorts with a controlled experimental design is warranted.

## 5. Conclusions

Longer telomeres and higher levels of pro- and anti-inflammatory cytokines were identified in athletes who belong to HI sports, suggesting less aging and a potentially healthier phenotype compared to athletes who belong to low- and moderate-intensity sports. Investigations of the functional significance of these associations on the health and performance of elite athletes and the use of TNF-α, IL-6 and IL-10 as potential biomarkers for aging-related changes in immune response in elite athletes are needed.

## Figures and Tables

**Table 1:** Classification of participants. Participating elite athletes (males: M and females: F) were divided into three groups depending on the intensity of their sports, and further dichotomized into In Competition and Out of Competition categories depending on timing of sample collection.

	Low Intensity (LI)	Moderate Intensity (MI)	High Intensity (HI)
In Competition	1 Cricket (M), 1 Equestrian (M), 1 Golf (M), 1 Powerboating (M), 1 Sport climbing (M)	4 Football (4M)	2 Cycling—track endurance (2M), 4 Cycling—cross (2M, 2F), 5 Triathlon (4M, 1F)
Out of Competition	1 Athletics—throws (M), 2 Bobsleigh (1M, 1F), 2 Gymnastics (2F), 1 Luge (M), 2 Sport climbing (1M, 1F), 1 Volleyball (F), 4 Wrestling (4M)	27 Football (26M)	12 Athletics—long distance (11M, 1F), 4 Cycling—track endurance (2M, 2F), 2 Cycling—road (2M), 2 Triathlon (2M)

**Table 2:** Comparing cytokines, antioxidant enzymatic activities and telomere length (TL) in younger (under 25 years old) and older (above 25 years old) elite athletes who belong to different sport intensities (low, moderate and high).

Cohort		Under 25				Above 25				*p* Value			
Variables	All (*n* = 80)	Combined (*n* = 45)	Low (*n* = 12)	Moderate (*n* = 16)	High (*n* = 17)	Combined (*n* = 35)	Low (*n* = 6)	Moderate (*n* = 14)	High (*n* = 15)	Age (Combined)	Age (Intensity)	Intensity (Age)	Age × Intensity
Age (years)	26.4 (7.6)	21.0 (2.2)	20.67 (2.02)	22 (1.51)	20.35 (2.52)	33.5 (6)	33.8 (9.5)	33.2 (2.2)	33.7 (7.3)	***	***	NS	NS
IL-6 (pg/mL)	0.7 [0.4–1.4]	0.7 [0.4–1.1]	0.7 [0.5–0.7]	0.9 [0.7–0.9]	0.4 [0.4–0.9]	0.7 [0.4–3.4]	0.4 [0.3–0.4]	0.5 [0.4–0.6]	3.4 [1.7–4.2]	NS	NS	**	***
IL-8 (pg/mL)	6.9 [3.9–11.7]	6.4 [3.3–10.4]	7.7 [4.1–14.6]	6.7 [5.1–8.9]	5 [1.6–11]	8.1 [4.7–16.2]	5.8 [3.9–17.6]	8.3 [4.9–9.5]	9.1 [2–75.6]	NS	NS	NS	*
TNF-α (pg/mL)	1.6 [1.0–2.5]	1.4 [0.9–2]	1.8 [0.7–2.4]	1.4 [0.9–2.1]	1.3 [1–1.7]	2.1 [1.1–3.3]	1.4 [1.2–4]	2.1 [1.7–2.9]	2.1 [1–4.9]	**	*	NS	NS
IL-10 (pg/mL)	0.3 [0.2–0.5]	0.3 [0.2–0.5]	0.3 [0.3–0.4]	0.3 [0.2–0.7]	0.3 [0.2–0.8]	0.4 [0.2–1.2]	0.4 [0.2–1.3]	0.3 [0.2–0.4]	0.4 [0.2–29.7]	NS	NS	*	NS
TL length (kb)	7.2 (4.0)	7.5 (4.4)	5 (3.1)	8.6 (5)	8.3 (4.3)	6.7 (3.3)	6.6 (5.7)	6 (2.5)	7.3 (2.7)	NS	NS	*	NS
SOD (U/mL)	2.5 (0.1)	2.5 (0.07)	2.5 (0.1)	2.6 (0.1)	2.5 (0.1)	2.5 (0.05)	2.6 (0.03)	2.5 (0.05)	2.5 (0.05)	NS	NS	NS	NS
Catalase (U/mL)	0.2 (0.1)	0.16 (0.12)	0.2 (0.2)	0.1 (0.1)	0.2 (0.1)	0.18 (0.14)	0.2 (0.2)	0.2 (0.08)	0.2 (0.2)	NS	NS	NS	NS

Data are presented as mean (SD) for normally distributed data or median (IQR) for skewed data. Comparisons were performed using *t*-test, Wilcoxon–Mann–Whitney, 1-way ANOVA, or linear models as appropriate. Indicated *p* values are for the following comparisons: Age (combined) compares variables between the two age groups without correcting for intensity (*t*-test and Wilcoxon–Mann–Whitney). Age (intensity) compares variables between the two age groups after correcting for intensity (linear model). Intensity (age) compares intensity groups after correcting for age (linear model). Age x intensity indicates significant interactions between age and intensity for the tested variables (linear model). * *p* ≤ 0.05, ** *p* ≤ 0.01, *** *p* ≤ 0.001. One unit of catalase decomposes one micromole of hydrogen peroxide per minute at 25 °C and pH 7.0. One unit of SOD is defined as the amount of enzyme causing half the maximum inhibition of the reduction of 1.5 mM Nitro blue tetrazolium in the presence of riboflavin at 25 °C and pH 7.8. TNF-α levels were higher in older elite athletes regardless of intensity groups: When comparing cytokine levels between athletes who belong to the two age groups, TNF-α was the only cytokine that showed a significant increase (*p* < 0.01) in older compared to younger elite athletes regardless of intensity (Figure 2A).

## Data Availability

Data are available from the corresponding author upon reasonable request.
